# Challenges to studying the health effects of early life environmental chemical exposures on children’s health

**DOI:** 10.1371/journal.pbio.2002800

**Published:** 2017-12-19

**Authors:** Joseph M. Braun, Kimberly Gray

**Affiliations:** 1 Department of Epidemiology, Brown University, Providence, Rhode Island, United States of America; 2 National Institutes of Environmental Health Sciences, Durham, North Carolina, United States of America; National Institute of Environmental Health Sciences, United States of America

## Abstract

Epidemiological studies play an important role in quantifying how early life environmental chemical exposures influence the risk of childhood diseases. These studies face at least four major challenges that can produce noise when trying to identify signals of associations between chemical exposure and childhood health. Challenges include accurately estimating chemical exposure, confounding from causes of both exposure and disease, identifying periods of heightened vulnerability to chemical exposures, and determining the effects of chemical mixtures. We provide recommendations that will aid in identifying these signals with more precision.

This Perspective is part of the *Challenges in Environmental Health: Closing the Gap between Evidence and Regulations Collection*.

## Introduction

Environmental chemical exposures are ubiquitous yet largely invisible. In the United States, over 85,000 chemicals are used in commerce, and thousands of these are produced in quantities of over one million pounds per year [1]. During our lives, we are exposed to many known toxicants, as well as numerous potentially hazardous chemicals with less well-characterized risks. These chemicals are detected in the blood and urine of almost every person in the US, as well as people in other countries [2]. Broad generalizations about these chemicals are difficult, but they can be categorized based on their uses in commerce (e.g., pesticides), routes of exposure (e.g., drinking water), toxicological effects (e.g., neurotoxicity), or persistence in biological tissues or the environment (e.g., long half-lives).

The potential toxicity of the vast majority of these chemicals is not routinely evaluated before they are introduced into commerce or industry [3]. While recent US legislation mandates assessments of the health effects of the most concerning of these chemicals [4], the scale of this problem is daunting given the large number of chemicals used and wide range of potential effects they could have on human health and development. For instance, >200 chemicals used in commerce or industry are known to be neurotoxic to humans, and approximately two new human neurotoxicants have been identified each year between 2006 and 2013 [3].

Quantifying the risk that chemicals pose to human health is of great interest to scientists, the public, policy makers, and regulators. Studies using laboratory animals, in vitro models, high-throughput screening, and human populations all provide valuable information when assessing the risks that chemicals pose to human health. Epidemiological studies provide estimates of the health risks of chemical exposures in human populations and primarily rely on observational data, as it would be unethical to experimentally assign chemical exposures to humans.

Epidemiological studies are not without challenges, and these challenges can be thought of as a signal-to-noise ratio problem. Epidemiologists must identify the “signals” of environmental chemical exposures from the “noise” that accompanies the observation of humans in their natural environments. In this commentary, we will discuss four major challenges faced by epidemiologists within the context of this signal-to-noise problem when estimating how environmental chemical exposures influence children’s health.

## Early life environmental chemical exposures and children’s health

There is particular concern that exposure to some chemicals during gestation, infancy, or childhood may increase the risk of obesity, asthma or allergies, or neurodevelopmental disorders [5]. These chemicals include pesticides (e.g., pyrethroids), naturally occuring metals (e.g., lead), and endocrine-disrupting chemicals (e.g., bisphenol A [BPA]).

Concern over chemical exposures during fetal, infant, and child development arises for several reasons. First, infants and children may have higher exposure to some chemicals than adults because they consume more water and greater quantities of specific foods, rely solely on breast milk or formula for the first months of life, and have higher ventilation rates, intestinal absorption, surface area-to-volume ratios, and hand-to-mouth activity [6]. In addition, the time-dependent and synchronized nature of their rapidly developing organ systems makes them more sensitive to environmental inputs that disrupt growth and development. Finally, the fetus, infant, and child may have higher chemical body burdens for a given dose of exposure because they have different pharmacokinetics compared to adults, which might alter the absorption and distribution of chemicals and decrease their capacity to metabolize and excrete chemicals.

Epidemiologists face at least four major challenges when estimating the potential effect of chemical exposures on child health; these include estimating chemical exposure, confounding, identifying periods of heightened vulnerablity, and chemical mixtures. Each of these challenges represents a source of noise in epidemiological studies that must be minimized in order to identify signals. Often, there is concern that this noise could result in the declaration of an adverse effect when one truly does not exist (i.e., false positives). However, it is equally important to note that noise could produce false negatives, where we miss the true effect of a chemical exposure.

## Estimating exposure to environmental chemicals

Measuring environmental chemical exposures in epidemiological studies requires valid and reliable assessment methods. Chemical exposures can be assessed through questionnaires, geospatial databases, environmental sampling, personal monitoring, or biomarkers. For example, pesticide exposure could be estimated using questionnaires about household pesticide use, pesticide release inventories in the vicinity of a person’s home, pesticide concentrations in household dust, pesticide concentrations in blood, or pesticide metabolites in urine.

Biomarkers provide objective and quantitative estimates of absorbed chemical exposure, and hundreds of chemicals can be sensitively and specifically measured in a variety of biospecimens, including blood, urine, breast milk, hair, and toenail clippings [7]. These biomarkers can include the exposure of interest or metabolites that are formed after ingestion. For instance, the organochlorine pesticide dichlorodiphenytrichloroethane is metabolized into dichlorodiphenyldichlorethylene, and concentrations of both can be measured in serum. While there is currently great enthusiasm for using biomarkers to assess chemical exposures, care must be taken to consider potential sources of bias in biomarker values, including differences in metabolism with disease state or age and exogenous contamination during sample collection, storage, processing, and analysis [8,9].

The choice of biomarker(s) for a given exposure depends on a number of factors, including the invasiveness of sample collection, sample storage/preparation/processing, and the pharmacokinetic properties of the exposure of interest [9]. Generally, chemicals that have long biological half-lives—on the order of months for lead and years for persistent chemicals—are measured in blood or serum and represent recent and past exposures. Chemicals with short biological half-lives—on the order of less than 24 hours for phthalates, phenols, and nonpersistent pesticides—are measured in urine and reflect exposure in the last few days.

**Fig 1:**
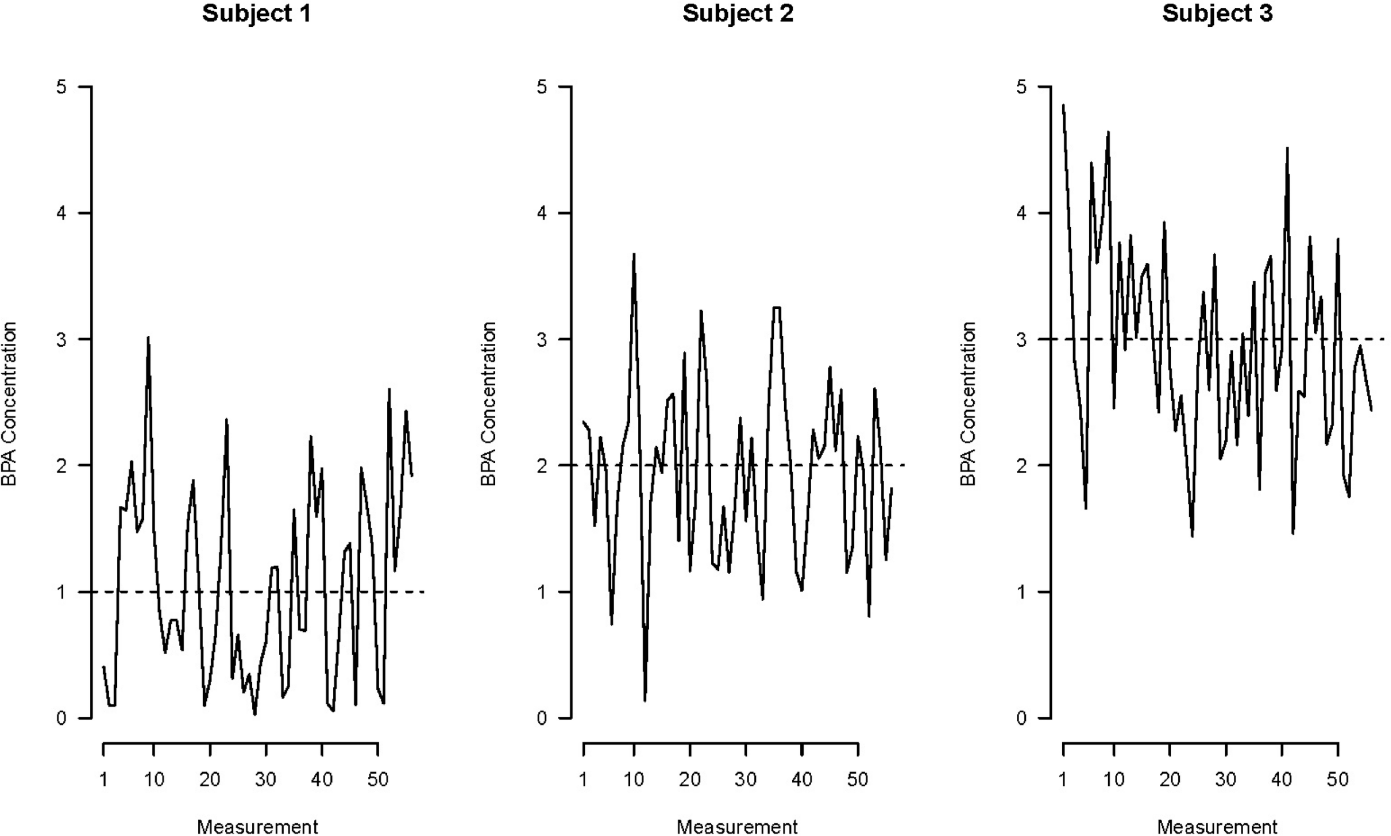
Urinary bisphenol A concentrations (ng/mL) from three hypothetical subjects over the course of approximately one week. Dashed lines represent average BPA concentration of all urine samples for each individual and were set arbitrarily to 1, 2, and 3 ng/mL. The x-axis denotes the sequential number of the collected urine sample. The figure illustrates that a single randomly chosen sample from an individual may not represent their average exposure and could misclassify them as having lower or higher exposure than their true exposure. Moreover, a single urine sample cannot reliably distinguish differences in BPA concentrations between individuals because the within-person variation in BPA concentrations is greater than the between-person variation. BPA, bisphenol A.

Errors in determining whether a person or population has been exposed to a given chemical, known as exposure misclassification, can distort links between exposure and outcomes. Assessing exposure to nonpersistent chemicals can be challenging because of their short biological half-lives and the episodic nature of exposure. For example, BPA is a potential endocrine disruptor with a biological half-life of approximately six hours that is found in some polycarbonate plastics and resins, food can linings, medical devices, and thermal receipts [10]. Nearly everyone in the US and many other countries is exposed to BPA [11]. Because of its short biological half-life and the variable nature of BPA exposure, there is considerable within-person variation in urinary BPA concentrations relative to differences between people. Thus, the difference in urinary BPA concentration from two individuals is more likely to reflect differences due to each individual’s own within-day variation in BPA exposure than differences in BPA exposure between the two individuals (Fig 1). When exposure misclassification occurs randomly among all people in a study regardless of disease outcome, then exposure misclassification is said to be nondifferential. Given that the basis of epidemiological studies is comparisons in health status between groups of individuals with different levels of exposure, nondifferential exposure misclassification creates noise in the data, drowning out potential signals and possibly creating false negative results.

It is important to note that exposure misclassification exists as a continuum and not as an all-or-none phenomenon. Thus, misclassification is generally more common with nonpersistent exposures arising from dietary sources than with nonpersistent exposures where the source of exposure is more stable over time. The latter includes chemical exposures found in personal care products (e.g., diethyl phthalate) or the indoor environment (e.g., butylbenzyl phthalate in flooring). Work by Perrier et al. has classified the magnitude of noise introduced by different levels of misclassification of nonpersistent chemical exposures [12]. They show that, for chemicals with substantial within-person variation (e.g., BPA), effect estimates derived from a study with a single urine sample can be attenuated by 80% and that as many as 35 repeated urine samples from a single individual are needed to reduce this attenuation to less than 10%. For chemicals with more moderate within-person misclassification (e.g., metabolites of diethyl phthalate), a single urine sample will result in effect estimates being attenuated by 40%, and six urine samples per person were required to reduce this attenuation to less than 10%.

One way to address the issues related to exposure misclassification of nonpersistent compounds is to pool multiple urine samples collected from individuals, as originally recommended by Perrier et al. and practiced by others [13]. This is a cost-effective solution, as it negates the need to conduct assays on dozens of urine samples and then take the average; instead, the pooled sample provides the arithmetic average of those samples while only requiring a single assay to be conducted per participant. Another potential solution is to measure concentrations of chemicals in shed deciduous teeth, toenail clippings, or hair [14]. These matrices are appealing when studying the health effects of chronic exposures, since some environmental agents, like heavy metals, accumulate in these slow-growing tissues over time and they can be noninvasively collected [15]. Thus, chemical concentrations in these matrices can provide a time-integrated exposure metric due to continuous incorporation of the chemical into the tissue.

## Finding the real causal actors

When conducting observational studies, it is possible that we misattribute the increased risk of disease to the environmental agent being studied when another factor that causes both exposure and disease risk is the real causal factor. This phenomenon, known as confounding, can arise when one or more determinants of health are also determinants of exposure. Such confounding factors may include age, race/ethnicity, biological sex, socioeconomic status, and diet. Many of these factors associated with disease risk are often also associated with environmental chemical exposures.

**Fig 2:**
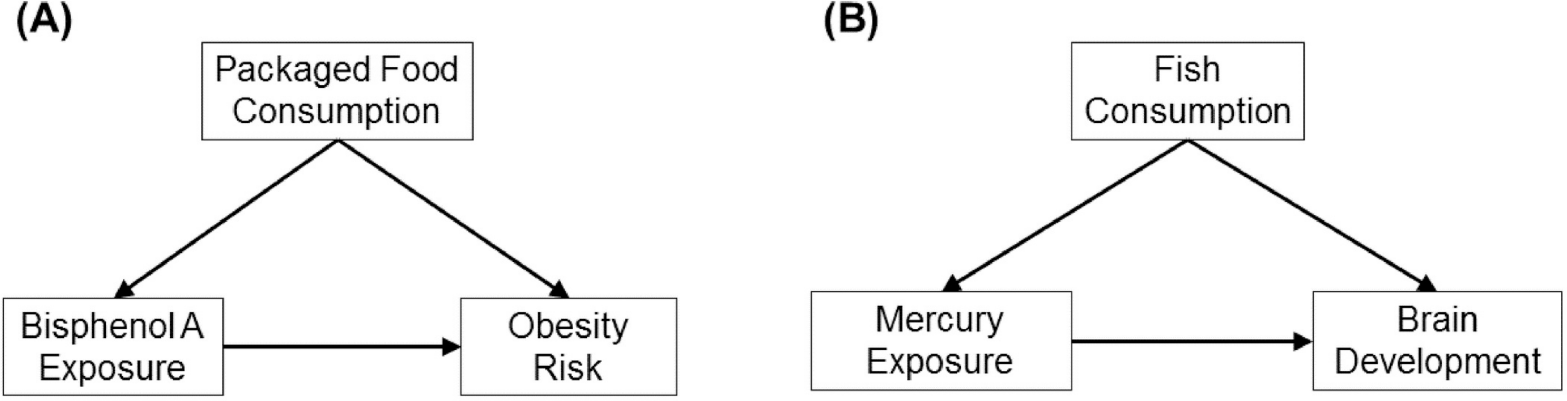
Hypothetical relations between (A) bisphenol A exposure, packaged food consumption, and obesity risk and (B) prenatal mercury exposure, fish consumption, and brain development. In panel A, a study is investigating whether bisphenol A is associated with increased risk of obesity and packaged food intake is associated with greater bisphenol A exposure and higher obesity risk. This is an example of positive confounding, where adjusting for packaged food consumption will cause the association between bisphenol A exposure and obesity to become weaker. In Panel B, we are investigating whether mercury exposure is associated with adverse brain development and fish consumption is associated with greater mercury exposure and better brain development. This is an example of negative confounding, and adjusting for fish consumption will make the association between mercury exposure and brain development stronger.

For example, suppose we observed that BPA exposure is associated with increased risk of childhood obesity. However, BPA is found in some food packaging and children who have higher risk of becoming obese might consume more packaged food that is less nutritious and more calorically dense than those children who do not consume as much packaged food (Fig 2A). Thus, failure to adjust for packaged food intake might lead us to falsely declare BPA a risk factor for obesity when the observation is really due to the calorically dense diet.

Though we are most concerned about confounding that artificially creates an association between an environmental chemical and child health that doesn’t truly exist, it is possible that we may fail to observe associations because of negative confounding. This less-appreciated form of confounding can arise when the confounding factor is associated with better health outcomes and higher levels of exposure. For example, prenatal mercury exposure was adversely associated with some aspects of child brain development, but only after adjusting for fish intake or serum polyunsaturated fatty acid concentrations during pregnacy (Fig 2B) [16,17]. This is because some fish are a source of both mercury and micronutrients that are beneficial to fetal brain development, and the effect of fish on both mercury and brain development obscured the effects of mercury on brain development. Negative confounding could also arise in studies of infant exposure to persistent chemicals in breast milk, as breast milk is a source of exposure to these chemicals and associated with many infant and child health outcomes.

It is imperative to note that it is not appropriate to adjust for variables that are both causes of exposure and childhood health. For instance, prenatal perfluoroalkyl substance (PFAS) exposures are associated with increased risk of childhood obesity [5]. Some might argue that it is necessary to adjust for birth weight, since birth weight is a determinant of childhood obesity risk [18]. However, since PFAS exposure is also associated with reduced birth weight, this adjustment will remove the effect that PFAS has on obesity through its association with birth weight. Thus, the association adjusted for birth weight will no longer reflect the total effect that PFAS has on the risk of obesity. In order to avoid this potential source of bias, confounding factors should be selected based on subject matter knowledge and not solely on statistical grounds.

Addressing confounding in epidemiological studies requires careful planning during the study design phase to ensure that important confounding factors are measured with valid and reliable instruments and are accounted for using appropriate methods [19]. Fortunately, most well-designed epidemiological studies studying environmental chemical exposures have carefully considered, collected, and adjusted for known confounding factors [20].

## Identifying periods of heightened vulnerability

The toxicity of some environmental chemicals may depend on the timing of exposure. The idea of discrete periods of heightened vulnerability has its origins in the study of teratogens, whose effects are present only when the exposure occurs during a specific period of fetal development [21]. One of the most infamous teratogens is thallidomide, a pharamaceutical agent used in the 1950s and 1960s to treat nausea in pregnant women. Thallidomide caused limb defects in thousands of children born to women who used the drug [22]. Notably, the presence of limb defects depended on the timing of thalidomide use, where exposure between 21 and 36 days after conception was necessary to cause these birth defects.

This notion of developmentally sensitive periods of development has been extended to include health outcomes that manifest later in life and is referred to as the Developmental Origins of Adult Health and Disease hypothesis [23]. One of the first examples of an exposure with both a discrete period of heightened vulnerability and long-term health effects was diethylstilbestrol (DES), a pharmaceutical given to women from the 1940s–1970s to prevent spontaneous abortion. Daughters born to women who were presecribed DES in the first half of their pregnany had increased risk of developing vaginal or cervical clear cell adenocarcinoma [24], as well as reproductive problems and some cancers [25].

The examples of thallidomide and DES highlight the challenges that epidemiological studies face when trying to identify periods of heightened vulnerability to early life chemical exposures. If there are discrete periods of vulnerability to chemical exposures during development, then this can be a source of noise, because researchers must measure exposure during that specific period in order to observe an association. Identifying periods of heightened vulnerability to a specific exposure can be challenging, as we often do not know if and when they exist. Thus, a lack of association between an environmental chemical exposure and child health may arise when exposure was not assessed during the etiologically relevant period.

While there has been justifiable emphasis on studying the toxicity of chemical exposures during fetal development, there are other potential periods of heightened vulnerability to environmental chemical exposure that likely depend on the specific chemical and health outcome of interest. An emerging body of evidence suggests that chemical and nonchemical exposures before conception may adversely affect the oocyte or sperm, which in turn may cause changes in the health status of the offspring via epigenetic reprogramming [26,27]. Periods of heightened vulnerability continue into infancy and childhood. For example, early childhood lead exposure is associated with reductions in cognitive abilites and increased risk of criminal arrests [28,29].

To identify periods of vulnerability, investigators need to assess exposure at multiple times during development, but this can be logistically challenging, especially for nonpersistent chemical exposures that require multiple biospecimens. Often, biospecimens are collected at times convenient for researchers and participants (e.g., routine clinic visits), but it is possible to have participants collect some biospecimens (e.g., urine) at their convenience and return them to researchers [30]. In addition, assessing exposure during some periods of development is difficult, like before conception or during early pregnancy when women may not know that they are pregnant. However, enrolling couples trying to conceive may help overcome this limitation [31]. A variety of statistical methods have been developed to identify unique periods of vulnerability to environmental exposures, even when these exposures are sparsely sampled during the period of interest [32]. Finally, we need to consider and jointly examine multiple periods of heightened vulnerability beginning before conception and continuing into adolescence and beyond.

## Chemical mixtures

Chemical exposures do not occur in isolation, and individuals are exposed to multiple chemicals on a daily basis across the lifespan. Most epidemiological studies have examined these exposures using a “one chemical at a time” approach that treats exposures as if they occur in isolation from each other. However, this does not reflect the nature of exposure to a multitude of chemicals or the potential for chemical exposures to have cumulative or interactive effects on human health. Thus, identifying signals from chemical mixtures may improve our understanding of risk factors for childhood diseases.

Epidemiological studies can address three broad questions related to chemical mixtures [33]. These include isolating the effect of individual exposures, determining if there are cumulative effects of exposure, and identifying interactions between chemical exposures. Recent examples of these types of studies include one quantifying the association between prenatal exposure to 52 chemicals and children’s autistic behaviors [34]. Another study estimated the cumulative effect of dioxin exposure on pubertal development in boys [35]. Finally, a group of researchers identified synergistic effects of toxic metals on children’s brain development [36].

A recent workshop by the National Institutes of Environmental Health Sciences (NIEHS) brought together toxicologists, epidemiologists, biostatisticians, regulators, and exposure scientists to develop new methods to improve our understanding of how chemical mixtures affect human health [37]. This workshop highlighted the innovative work being done by multiple groups of investigators and showed that there is great promise to apply these methods to existing studies in the hopes of identifying new risk factors for diseases and developing more targeted public health interventions. However, it is still unclear which of the many methods presented are best at addressing specific chemical mixture questions, and more work is needed to compare these methods to one another in different populations and under different assumptions. Moreover, it is unclear if complex and sophisticated methods provide better estimates of risk than more traditional methods. The NIEHS is addressing this by supporting innovative research to develop and disseminate new methods for mixtures research using the recently announced Powering Research through Innovative Methods for mixtures Epidemiology (PRIME) Program (RFA-ES-17-001, https://grants.nih.gov/grants/guide/rfa-files/RFA-ES-17-001.html). Forthcoming studies from this research initiative have the potential to improve our detection of signals from environmental chemical mixtures.

## Conclusions

Epidemiological studies face challenges related to measuring chemical exposures, confounding, periods of heightened vulnerability, and mixtures when identifying the effect of environmental chemicals on children’s health. We provide several recommendations related to each of these points in Table 1 that we believe will enhance our ability to identify the signals of early life chemical exposures with more precision. There will be a continued need to use existing studies to investigate the long-term effects of early life chemical exposures, and the implementation of our recommendations in new cohorts will help us identify the potential health effects of emerging chemicals of concern.

**Table 1:** Recommendations to improve the design and analysis of studies examining environmental chemical exposures and children’s health.

Challenge	Recommendations
Exposure Assessment	• Collect repeated urine samples from a person and pool the samples when assessing exposure to non-persistent chemicals • Use integrative biospecimens (e.g., teeth, hair, or toenails) when the exposure of interest can be accurately assessed in such matrices
Confounding	• Identify potential confounders before the onset of the study • Consider potential confounding from other chemicals or pollutants correlated with the exposure(s) of interest • Assess potential confounders with valid and reliable methods • Do not adjust for variables that are caused by the exposure and causes of the disease (e.g., causal intermediates)
Periods of Vulnerability	• Assess exposure at multiple times during gestation, infancy, and childhood • Consider assessing preconception maternal and paternal chemical exposures when feasible • Assess exposure as early as possible in pregnancy (i.e., during the first trimester) • Use appropriate statistical methods to identify periods of heightened vulnerability
Chemical Mixtures	• Consider what mixture-related question the study will address during the design phase • Collect appropriate biospecimens for analysis of target chemicals • Use appropriate statistical methods to address mixture-related question of interest

## Abbreviations

BPA: bisphenol A
DES: diethylstilbestrol
NIEHS: National Institutes of Environmental Health Sciences
PFAS: perfluoroalkyl substance
PRIME: Powering Research through Innovative Methods for mixtures Epidemiology
